# Clinical Value Analysis of Combined Vaginal Ultrasound, Magnetic Resonance Dispersion Weighted Imaging, and Multilayer Spiral CT in the Diagnosis of Endometrial Cancer Using Deep VGG-16 AdaBoost Hybrid Classifier

**DOI:** 10.1155/2022/7677004

**Published:** 2022-04-26

**Authors:** Xiaoyi Wang, Rong Zhang

**Affiliations:** Department of Function, Changzhou Geriatric Hospital Affiliated to Suzhou University, Changzhou Seventh People's Hospital, Changzhou, 213000 Jiangsu, China

## Abstract

Endometrial carcinoma is one of the most common disorders of the female reproductive system. Every year, around 76,000 women die from endometrial cancer around the world. Endometrial cancer is a significant factor in women's health, particularly in industrialized nations, where the prevalence of this tumor type is the greatest. It is an important concern in women's health because of disease mortality and the rising number of new diagnoses. The aim of the study was to investigate the clinical value of combined transvaginal ultrasound, magnetic resonance dispersion weighted imaging, and multilayer spiral computed tomography (CT) in the diagnosis of early-stage endometrial cancer. Initially, the dataset is collected that consisted of a total of 100 cases and split into the control group and experimental group of 50 cases in each group. The control group is diagnosed using conventional Doppler ultrasound diagnostic machine. The experimental group is diagnosed with combined ultrasound method. The ultrasound images thus obtained are preprocessed using the speckle-free adaptive wiener filter. The preprocessed images are segmented using the fuzzy clustering segmentation method. The features are extracted by the independent component analysis (ICA) method. We have proposed the deep VGG-16 AdaBoost hybrid classifier for classifying the normal and abnormal images. The clinical value of the diagnosis is analyzed using the parameters like diagnostic accuracy, specificity, sensitivity, and kappa coefficient. It is observed that the clinical value is better for the experimental group than the control group.

## 1. Introduction

Endometrioid histology is the most frequent kind of endometrial cancer, accounting for 80% of all cases. In general, endometrioid endometrial cancer carcinogenesis is preceded by complicated atypical hyperplasia.

Postmenopausal bleeding (PMB) is a frequent occurrence that affects around 10% of postmenopausal women. While it is necessary to rule out endometrial cancer (EC) during the diagnostic process, most PMB has a benign origin, and only about 10% of women with PMB will be diagnosed with cancer. Nonetheless, timely treatment is critical since patients with localized EC have a five-year survival rate of over 95 percent, but patients with regional and distant metastases have survival rates of around 70 percent and 20 percent, correspondingly [1]. Endometrial carcinoma refers to a group of epidermal malignant tumors that grow in the endometrial, the most frequent of which is adenocarcinoma, which originates in the endometrial glands. Furthermore, EC often manifests itself in postmenopausal and perimenopausal women. There are over 200,000 new instances of ovarian cancer every year [2, 3], making it one of the most prevalent cancers of the female reproductive system. After ovarian cancer and cervical cancer, it is the third most deadly gynaecological malignant tumor. With the fast expansion of China's social economy, the incidence of EC is on the rise, and it now ranks second among malignant tumors. EC's cause has not been determined at this point. A number of studies have connected it to a person's fertility, metabolism, hormones, and behavior. In the endometrium, endometrial hyperplasia is a pathological change that may lead to cancer and may deteriorate. Atypical endometrial hyperplasia has a morphology that is quite similar to EC, making clinical identification challenging. Given the risk of endometrial hyperplasia and the difficulties of clinical diagnosis, a technique that can intelligently distinguish between normal endometrium, endometrial hyperplasia, and endometrial malignant cells is required [4–7].

A malignant tumor in the female reproductive system, endometrial cancer accounts for around 25 percent of all female reproductive system malignancies, and it is more common in developed countries than in developing ones.

EC is common in perimenopausal and postmenopausal women, and a recent study found that the age of patients when they first get endometrial cancer is becoming less and lower. Despite the fact that the five-year survival rate of patients has increased as medical technology has advanced, the prognosis of certain EC patients is poor, resulting in mortality; consequently, early detection of endometrial cancer patients is critical. Patients with advanced endometrial cancer have a substantially greater death risk than those with early stage EC. An important step in improving patient survival rates is increasing the specificity and sensitivity of EC diagnosis [8–11]. In the diagnosis of cancers, ultrasound plays a critical role. Ultrasound provides a number of several benefits, including noninvasiveness, high diagnostic sensitivity, lack of radioactivity, and straightforward techniques. It is often utilized in tumor diagnostics. Furthermore, via its early screening, ultrasonography may minimize the number of invasive examinations for patients as well as the dangers of invasive surgery. T-cell-activating protein (TAP) is an aberrant glycoprotein and calmodulin complex that is produced in cells when oncogenes and tumor suppressor genes are mutated. In this paper, we offer a novel deep blended learning for endometrial cancer detection, which uses a pretrained deep neural network combined with the AdaBoost classifier. AdaBoost is the first effective boosting algorithm for binary classification tasks, and it learns by weighting training examples and weak learners and then predicts by weighting predictions from weak learners. Furthermore, we assessed accuracy using a variety of statistical indicators. Despite the introduction of many new and better scoring models since VGG was first suggested, VGG16 continues to pique the curiosity of data scientists and researchers throughout the world. VGG16 can be utilized in medical imaging, such as X-ray or MRI, to diagnose disease. Cancer incidence, development, invasion, metastasis, and prognosis are all linked to aberrant glycoproteins, according to studies. As a result, detecting TAP in serum may be useful for clinical tumor diagnosis [12–15]. The other sections of the article are arranged as follows: Section 2 goes into great depth regarding the suggested work flow, Section 3 goes through the results and discussion, and Section 4 concludes up the study.

## 2. Materials and Methods

**Figure 1:**
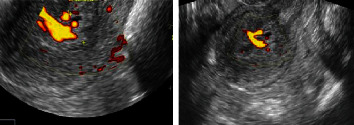
Transvaginal ultrasound screening.

In this research, we look at EC and the whole work flow shown in Figure 1. The speckle-free adaptive wiener filter is used to preprocess the ultrasound pictures acquired in this way. The fuzzy clustering segmentation technique is used to segment the preprocessed pictures. The independent component analysis (ICA) approach is used to extract the features. For categorizing normal and abnormal pictures, we suggested the deep VGG-16 AdaBoost hybrid classifier.

All studies were in compliance with the Declaration of Helsinki and applicable regulatory requirements. This study was approved by the Research Ethics Committee of Changzhou geriatric hospital Affiliated to Suzhou University, Changzhou Seventh People's Hospital.

### 2.1. Dataset Collection

A total of 100 women with probable EC who were hospitalized to the Department of Function, Changzhou geriatric hospital affiliated to Suzhou University between September 2018 and September 2020 were chosen and randomly split into the control group (images = 50) and experimental group (images = 50). The control group had a conventional ultrasound examination; the observation group received TAP in addition to conventional ultrasound examination, and all patients were definitively identified by hysteroscopy and diagnostic curettage examination. Patients were excluded if they had a history of tumors, had liver, kidney, or other organ malfunction, or had abnormal bleeding or coagulation dysfunction prior to surgery. Patients with unqualified curettage specimens, those who had already received therapy, those who had a significant tumor mass, and those who had additional pulmonary or chest wall disorders were also eliminated [16]. Changzhou Seventh People's Hospital's Ethics Committee authorized the trial, and patients or their family members provided in formed permission.

### 2.2. Conventional Ultrasound Using Doppler Ultra Sound Diagnostic Machine

Using a Doppler ultrasonography diagnostic equipment, power Doppler analyzes endometrial volume and vascular indicators such as the vascularization index (VI), flow index (FI), and VFI vascular flow index (VFI) in 50 women. These indicators indicate to tumor neovascularization, boosting the risk of cancer. In addition, examining the vascular patterns might reveal more about the tumor's biology [17]. Several studies have shown that power Doppler can help distinguish between malignant and benign endometrial lesions, but only one study has shown a comprehensive correlation among power Doppler indices and endometrial histological types, grade, myometrial and cervical invasion, and lymph vascular space invasion.

### 2.3. Combined Ultrasound

- Transvaginal ultra sound: Figure 1 illustrates the transvaginal ultrasound. In women with PMB, transvaginal ultrasonography is a very accurate approach for diagnosing EC. It might indicate the kind of endometrial abnormalities, such as endometrial hyperplasia, polyps, or cancer. If the endometrium is thickening diffusely or focally, a sample of the endometrium must be taken. Because the ultrasound appearances overlap so much, pathological confirmation of the histology is required in all instances. Ovarian disease, such as polycystic ovaries in younger women and ovarian tumors secreting estrogens that cause aberrant vaginal bleeding, may also be detected using transvaginal ultrasonography [18]

(ii) Magnetic resonance dispersion weighted imaging: MRI is a powerful imaging technology that can help women with endometrial cancer receive treatment based on a multidisciplinary team's consensus. In the staging MRI, the depth of myometrial invasion, cervical extension, and lymph node metastases is all important. The use of MRI aids in the correct planning of treatment and the identification of patients who will benefit from para-aortic lymph node dissection. The imaging and clinical aspects of the two primary forms of endometrial cancer must be understood by radiologists. For endometrial cancer staging, imaging techniques can be refined, and sophisticated techniques can increase the accuracy of local tumor spread. When compared to normal myometrium, endometrial cancer is often moderately hyperintense on T2-WI. Subtype II has an inhomogeneous morphology with areas of bleeding and necrosis and is frequently diagnosed as a result of deep myometrial invasion. Exophytic expansion is seen in the majority of tumors that develop from the fundus. Diffuse infiltrative growth, which is characterized by diffuse myometrial thickening, is uncommon

To determine the depth of myometrial invasion, the uterus must be evaluated in at least two planes using T2WI (maximal 4 mm slice thickness) along the uterine axis.

**Figure 2:**
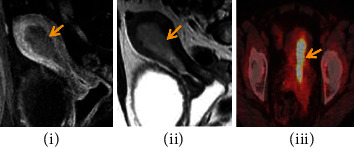
Magnetic resonance dispersion weighted analysis.

For pretreatment local staging of EC, MRI is the most accurate imaging modality because to its outstanding soft-tissue delineation capabilities. If detected by MRI, an endometrial cyst is often shown as a hypo-to-isointense T1-weighted mass with a signal intensity intermediate to that of a normal endometrium on T2 weighted scans. As demonstrated in Figure 2, the EC increases less than the myometrium [19].

(iii) Multilayer spiral CT: CT scans are less useful for examining uterine abnormalities

**Figure 3:**
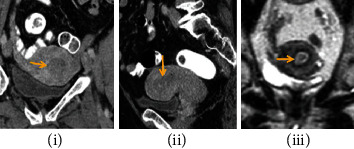
Multilayer spiral CT analysis.

CT, on the other hand, provides a higher multiplanar spatial resolution that can be used to visualize the entire pelvic and abdominal cavities for enlarged nodes and gross soft tissue masses, as well as distant lung metastases. It is demonstrated in Figure 3 that on contrast-enhanced CT, the EC is hypoattenuating and hypoenhancing. However, the CT appearance of a hypoenhancing endometrial mass is generic, and the possible diagnoses include submucosal leiomyomas, endometrial polyps, or cervical stenosis [20].

### 2.4. Image Preprocessing Using Speckle-Free Adaptive Wiener Filter

**Figure 4:**
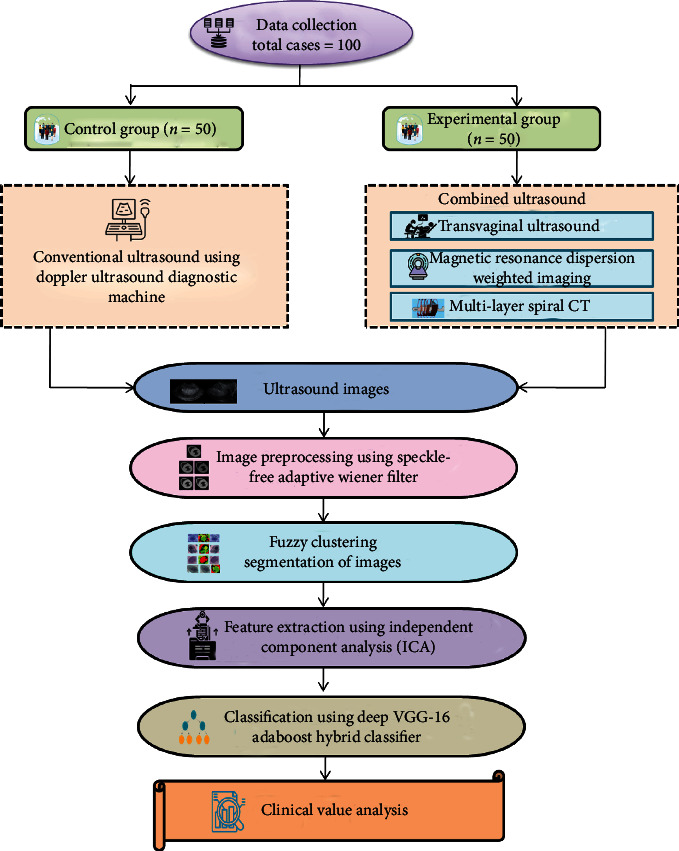
Overall proposed flow diagram.

In order to get the best estimation of the signal from a noisy temporal order, a statistical filter called the speckle-free adaptive wiener filter is used (Figure 4). In order to deal with speckle noise, we use adaptive Wiener filter. Gaussian Markov generalized additive noise reduction may be achieved by altering the kernel of the classic adaptive WF. In [21] approach to preserve edge and detail while reducing noise, the target contained in the noise is provided by the reference target.

(1) $$\begin{array}{c} O\left(i,j\right)=R\left(i,j\right)+m\left(i,j\right). \end{array}$$

Here, *O*(*i*, *j*) denotes original image, *R*(*i*, *j*) denotes reference object, and *m*(*i*, *j*) denotes noise.

In addition, the adaptive wiener filter for restoring the noise is provided by

(2) $$\begin{array}{c} H_{J}\left(i,j\right)=\frac{{\left|J\left(i,j\right)\right|}^{2}}{{\left|J\left(i,j\right)\right|}^{2}+{\left|M\left(i,j\right)\right|}^{2}}. \end{array}$$

Here, *J* (*i*, *j*) and *M* (*i*, *j*) represent Fourier transformations of *R*(*i*, *j*) and *m*(*i*, *j*) correspondingly. The next step is to identify the images' hidden targets. The image recognition plan is as follows:

(3) $$\begin{array}{c} H_{Jp}\left(i,j\right)=\frac{J{\left(i,j\right)}^{*}}{{\left|J\left(i,j\right)\right|}^{2}+{\left|M\left(i,j\right)\right|}^{2}}. \end{array}$$

Pattern recognition may be divided into a restoration AWF with an inverse filter, as seen in the following:

(4) $$\begin{array}{c} H_{Jp}\left(i,j\right)=\frac{1}{J\left(i,j\right)}\frac{{\left|J\left(i,j\right)\right|}^{2}}{{\left|J\left(i,j\right)\right|}^{2}+{\left|M\left(i,j\right)\right|}^{2}},\\ H_{Jp}\left(i,j\right)=\frac{1}{J\left(i,j\right)}H_{J}\left(i,j\right). \end{array}$$

The complex conjugates are denoted by an asterisk.

### 2.5. Fuzzy Clustering Segmentation of Images

The fuzzy clustering technique is used to segment the images. Fuzzy clustering algorithms are used to detect commonalities between pixels or a cluster of pixels in order to uncover unique structures in the picture feature space [22]. Furthermore, it divides a group of pixels from the input picture feature space into a set of homogenous pixels. Furthermore, one of the unsupervised clustering approaches is fuzzy clustering. Because of its simplicity, this is the most often utilized approach. The fuzzy clustering segmentation is used to discover an image partition with “*f*” fuzzy clusters for a set of segmentation sj ∈ *Q*, *i* = 1, 2,3,..., *C* while minimizing the cost function.

(5) $$\begin{array}{c} I\left(V,N\right)=\overset{f}{\underset{j=1}{\sum }}\overset{C}{\underset{i=1}{\sum }}{\left(V_{j,i}\right)}^{n}d_{j,i}, \end{array}$$

where *V* = [*V*_*j*_, *i*] represents as matrix of fuzzy partition.

The participation coefficient of the *i*-th picture object in the *j*-th clusters is *V*_*j*_, *i* ∈ [1, ∞].

*N* = [n1, ..⋯nf] is denoted as centre matrix clustering.

Furthermore, the word *N* [1] is mentioned as a fuzzification parameter that employs the conventional distance metric known as Euclidean distance to determine the distance between the areas *y*_*i*_ and *n*_*j*_. The suggested fuzzy cluster-based segmentation method operates in the following phases:

- Set the appropriate values for variables like *n* and *c*, as well as a tiny positive integer
- For the time period *y*_1_ and *y*_2__,_ pick the cluster center at random
- Assume that the parameter *y* = 0
- For the time intervals *y*_1_ and *y*_2_, calculate the fuzzy value of the partition matrix at *y* = 0
- Make the *y* > 0 value and the fuzzy division matrix *v* as follows:

(6) $$\begin{array}{c} v_{j,i}^{\left(y+1\right)}=\frac{1}{\left(\sum_{l=1}^{f}{\left(d_{j,i}/d_{j,i}\right)}^{1/\left(1-m\right)}\right)}\left(y_{1},y_{2}\right), \end{array}$$

for *j* = 1, ⋯., *f* and *i* = 1, ⋯., *C*.

Update the *y* = 0 or verify that the requirement is met:

(7) $$\begin{array}{c} \left\|N^{\left(y+1\right)}-N^{\left(y\right)}\right\|\left(y_{1},y_{2}\right)<\varepsilon . \end{array}$$

The clustering technique here takes into account the three groupings.

### 2.6. Feature Extraction Using Independent Component Analysis

Suppose that we observe a *K*-dimensional zero mean input pattern at time *l*, *a* (*l*) = [*a*1 (*l*), *a*2 (l)..⋯, ak (*l*)], where VT means the transposition of matrices and vectors. Assume that this pattern *a* (*l*) is linearly composed of *N* statistically independent components *s*(*l*) = [*s*1(*l*), *s*2(*l*),. ⋯ ⋯*sN*(*l*)]^VT^ (*N* < *K*) as shown below:

(8) $$\begin{array}{c} a\left(l\right)=Xs\left(l\right). \end{array}$$

Here, *X* means a *N* × *K* mixing unknown matrix. We calculate *X* in such a way that each element of *s* (*l*) is as independent as feasible. Initially, PCA is used to whiten the input sequence *s* (*l*) in this approach, as stated in the following expression.

(9) $$\begin{array}{c} u\left(l\right)=D^{-1/2}V^{P}a\left(l\right). \end{array}$$

Here, *D* is denoted as diag [*β*i,.....*β*K], and *V* represents [v1, v2, v3,........vk], where *β*i represents as the *i*-th biggest eigenvalue of the covariance matrix, and vi denotes as *i*-th eigen vector. The uncorrelated nature of characteristics is a need for independence. As a result, higher-order statistics elements are often uncorrelated after whitening. The following equation may be used to calculate independent components $\tilde{s}\left(l\right)$ using *u*(*l*):

(10) $$\begin{array}{c} \tilde{s}\left(l\right)=Qu\left(l\right). \end{array}$$

Here *Q* is an *N* × *N* a separation matrix with the constraint of being orthonormal.

The input-output relationship in the independent component analysis (ICA) procedure is expressed as follows by Equations (9) and (10):

(11) $$\begin{array}{c} \tilde{s}\left(l\right)={QD}^{-1/2}V^{P}a\left(l\right)=Ba\left(l\right). \end{array}$$

The following equation represents *Q*'s update formula in the proposed ICA algorithm:

(12) $$\begin{array}{c} Q_{l+1}=Q_{l}+\beta \left(\tanh\;\tilde{\;s}\left(l\right)\right)u{\left(l\right)}^{P}+\gamma Q_{l}\left(I-Q_{l}Q_{l}^{P}\right)Q_{l}. \end{array}$$

### 2.7. Deep VGG-16 AdaBoost Hybrid Classifier

(13) $$\begin{array}{c} G\left(y\right)=\text{sign}\left\{\overset{Q}{\underset{q=1}{\sum }}\propto_{q}g_{q}\left(y\right)\right\}, \end{array}$$

where *y* represents input vector; *g*_*q*_(*y*), *q* = 1,…, *Q* suggests that there are a lot of classifiers which is *Q*; *g*_*q*_, *q* = 1,....., and *Q* represents each weak classifier; the weight, suppose a training data collection {*r*_*j*_, *s*_*j*_}, *j* = 1,…, *n*, where *r*_*j*_ ∈ *T*^*n*^,  *s*_*j*_∈ {1, -1}, is provided and to apply the categorization results and maintain a maximum identification rate and makes the dispersion values of a positive data equal to that of all negative values at the start of the process; if the training data collection contains *p* positive data and *q* negative data, that is, *n* = *x* + *y*, we set the dispersion value of the positive data and that of the negative data as 1/(*x* + 1) and 1/*y*(*x* + 1), respectively, and deep VGG-16 and the selection loop for *T* weak classifiers are then performed by AdaBoost classifier for classifying EC detection; every time the algorithm conducts the loop, it first looks for weak classifiers *g*_*q*_ (*y*), *q* = 1,......, *n* with minimized inaccuracy in each dimension and then chooses the weak classifier *g*_*q*_ (*y*) among these weak classifiers with the least error; Realign the probability value of the training set after choosing weak classifiers at *t* cycle so that samples of categorization error in this cycle have precedence when the further cycle is run, then compute the relevant weight ∝_*q*_ of the weak classifier *g*_*q*_(y), lastly, calculate the total of products of weights of *T* denotes weak classifiers and equivalent ∝values to get the strong classifier *G* (*y*) and calculate the categorization outcome of the input vector with the ensemble classifier outcome.

## 3. Performance Analysis

TAP is a compound of aberrant glycoproteins, calcium-histone, and the common material that genes express once cancer cells have developed. As a result, TAP can be utilized to infer the number and severity of malignant cells. When tumor cells reach a specific size, a high number of these chemicals are released into the bloodstream, where they can be detected. TAP examination is a particular identifying procedure that uses a coagulant to help tumor aberrant protein aggregation and the creation of certain crystalline particles. Human blood samples without TAP material, on the other hand, cannot crystallize. A multistage coupling condensation reaction is used in the TAP examination technology. To begin, primary condensates are formed by mixing various coagulants with various aberrant sugar chain glycoproteins. The same or different primary condensates then clump together to create secondary condensates linked by calcium-histone, which are the condensed particles seen in TAP analysis.

The performance analysis for both the control and experiment group was analyzed. The procedure for EC diagnosis for both the control and experimental group is identical. The experimental group using combined ultrasound such as transvaginal ultrasound, magnetic resonance dispersion weighed imaging, and multilayer spiral CT has the better performance compared to the control group utilizing the conventional Doppler ultrasound technique. Tables 1 and 2 indicate the comparison of various algorithms for the control group and the experiment group.

**Figure 5:**
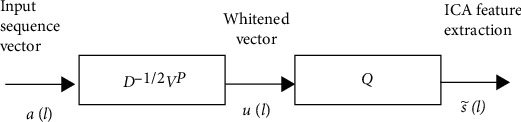
General block diagram for ICA feature extraction.

- The accuracy refers to the cases that were successfully categorized for the provided test data set, as shown in Figure 5. The findings show that while the number of training data is low (10% and 20%), the AdaBoost method without feature selection performs better, but when the number of training data is high (30% to 80%), the decrease of attributes enhances prediction accuracy. In comparison to other algorithms, the AdaBoost algorithm outperforms them all:

(14) $$\begin{array}{c} \text{Accuracy}=\frac{\text{TP}+\text{TN}}{\text{TP}+\text{FP}+\text{TN}+\text{FN}}. \end{array}$$

**Figure 6:**
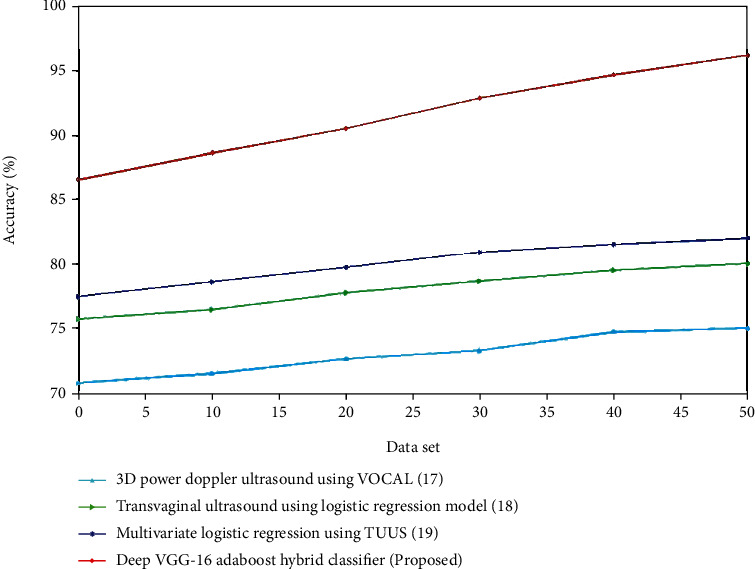
Comparison of accuracy (%) for existing and proposed methods.

**Figure 7:**
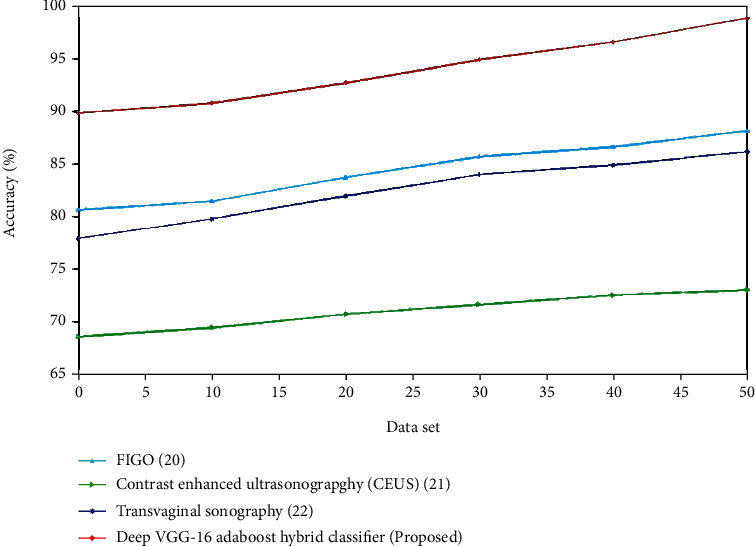
Comparison of accuracy (%) for existing and proposed methods.

Figures 6 and 7 present a comparison of accuracy among the present and suggested procedures for the control and experimental groups. The graph clearly demonstrates that the new technique is more accurate than the existing ones. For the control group, the accuracy of transvaginal ultrasound using the logistic regression model is lower compared to the multivariate logistic regression using TVUS. The deep VGG-16 AdaBoost hybrid classifier has high accuracy compared to the 3D Doppler ultrasound using VOCAL, transvaginal ultrasound using logistic regression model, and multivariate logistic regression using TVUS. For the experimental group, the accuracy of contrast-enhanced ultrasonography is lower compared to the FIGO. The deep VGG-16 AdaBoost hybrid classifier has the greater accuracy value compared to the FIGO, transvaginal sonography, and contrast-enhanced ultrasonography.

(ii) Sensitivity: improved diagnostic performance appears to be beneficial for improved risk classification in EC patients. The improved sensitivity is almost certainly due to the inclusion of more texture features in the evaluation.

(15) $$\begin{array}{c} \text{Sensitivity}=\frac{\text{TN}}{\text{TN}+\text{FN}}. \end{array}$$

**Figure 8:**
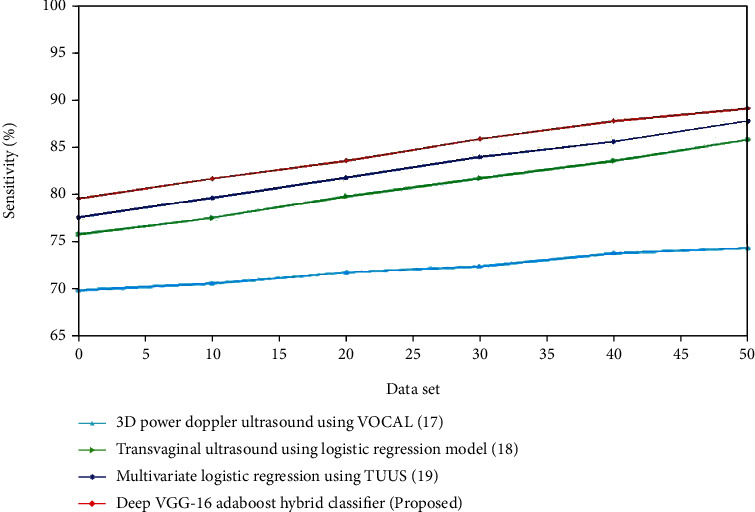
Comparison of sensitivity (%) for the existing and proposed method.

**Figure 9:**
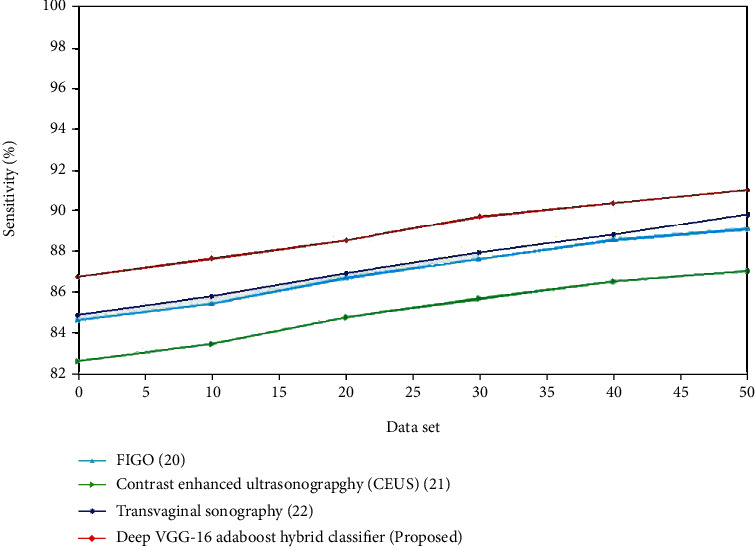
Comparison of sensitivity (%) for the existing and proposed method.

The assessment of sensitivity for the existing and suggested approaches is shown in Figures 8 and 9. The recommended method outperforms the existing methods. For the control group, the sensitivity of transvaginal ultrasound using the logistic regression model is lower compared to the multivariate logistic regression using TVUS. The deep VGG-16 AdaBoost hybrid classifier has high accuracy compared to the 3D Doppler ultrasound using VOCAL, transvaginal ultrasound using the logistic regression model and multivariate logistic regression using TVUS. For the experimental group, the sensitivity of contrast-enhanced ultrasonography is lower compared to the FIGO. The deep VGG-16 AdaBoost hybrid classifier has the greater sensitivity value compared to the FIGO, transvaginal sonography, and contrast-enhanced ultrasonography.

(iii) Specificity: endometrial thicknesses as a predictor of EC diagnosis had a much higher AUC (area under the curve) than random assignment. On the other hand, the ROC curve demonstrates that none of the cutoff points offered the optimum diagnostic results in terms of combining higher sensitivity with appropriate specificity rates, as needed by clinical practice

(16) $$\begin{array}{c} \text{Specificity}=\frac{\text{TN}}{\text{TN}+\text{FP}}. \end{array}$$

**Figure 10:**
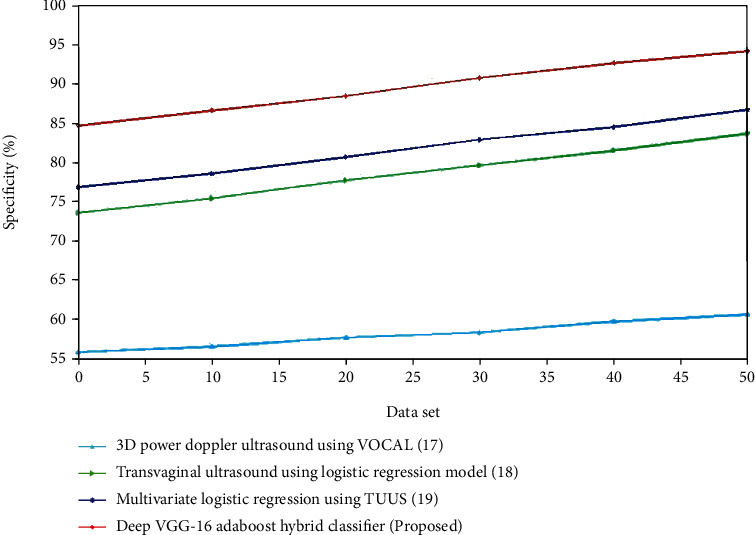
Comparison of specificity (%) for the existing and proposed method.

**Figure 11:**
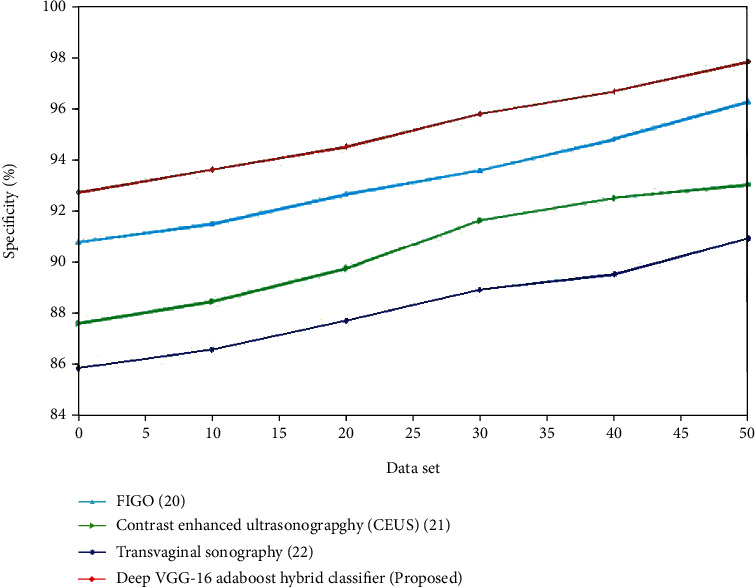
Comparison of specificity (%) for the existing and proposed method.

The evaluation of sensitivity for the present and proposed approaches is shown in Figures 10 and 11. The recommended method outperforms the existing methods. For the control group, the specificity of transvaginal ultrasound using the logistic regression model is lower compared to the multivariate logistic regression using TVUS. The deep VGG-16 AdaBoost hybrid classifier has high specificity compared to the 3D Doppler ultrasound using VOCAL, transvaginal ultrasound using logistic regression model and multivariate logistic regression using TVUS. For the experimental group, the specificity of contrast-enhanced ultrasonography is lower compared to the FIGO. The deep VGG-16 AdaBoost hybrid classifier has the greater specificity value compared to the FIGO, transvaginal sonography, and contrast-enhanced ultrasonography.

(iv) Kappa coefficient: the kappa statistic is a measure of how well anticipated and real classification in a dataset matches, with scores ranging from -1 to 1. A statistic correlation with a kappa statistic value higher than 0.7 is normally considered excellent, but the greater the number, the better the association. Reduces have the greatest kappa statistic of 0.90 and the lowest of 0.696, suggesting a strong to medium level of acceptance. When no reducts are employed, the kappa value scales from 0.702 to 0.889. When the AdaBoost method is employed to the decreased data set, the agreement of forecast represented by the kappa statistic is the highest, whereas it is least in the dataset without reductions.

**Figure 12:**
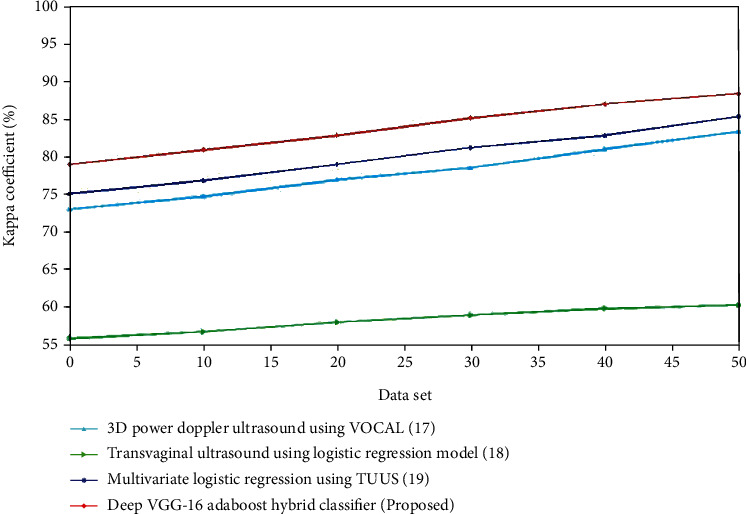
Comparison of specificity(%)for the existing and proposed method.

**Figure 13:**
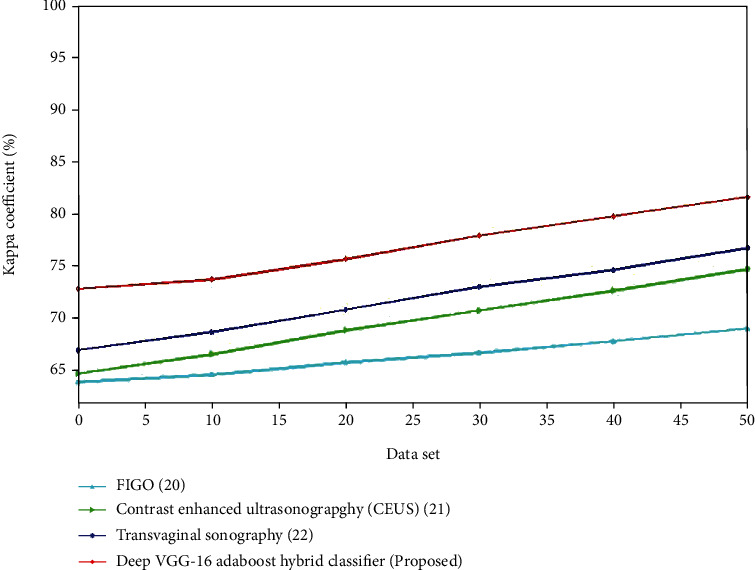
Comparison of specificity (%) for the existing and proposed method.

The comparison of sensitivity for the existing and suggested approaches is shown in Figures 12 and 13. The proposed technique gives efficient ones. For the control group, the kappa coefficient of transvaginal ultrasound using the logistic regression model is lower compared to the multivariate logistic regression using the TVUS. The deep VGG-16 AdaBoost hybrid classifier has high kappa coefficient compared to the 3D Doppler ultrasound using VOCAL, transvaginal ultrasound using the logistic regression model, and multivariate logistic regression using TVUS. For the experimental group, the kappa coefficient of contrast-enhanced ultrasonography is higher compared to the FIGO. The deep VGG-16 AdaBoost hybrid classifier has the greater kappa coefficient value compared to the FIGO, transvaginal sonography, and contrast-enhanced ultrasonography.

## 4. Conclusion

Early diagnosis of cancers in postmenopausal women who have vaginal bleeding is critical. There are already various detection methods available for identifying postmenopausal individuals with vaginal bleeding and estimating their risk of EC; however, not all healthcare practitioners agree on how to apply them. All postmenopausal women with EC and vaginal bleeding in the current study had an endometrial thickness equal to or higher than 5 mm on transvaginal ultrasonography. The dataset is divided into the control and experimental group. The conventional Doppler ultrasound techniques are used for the control group, whereas the combined ultrasound techniques such as transvaginal ultrasound, magnetic resonance dispersion weighted imaging, and multilayer spiral CT are utilized for the experimental group The result shows that the experimental group utilizing the combined ultrasound techniques has a better value for accuracy, specificity, sensitivity, and kappa coefficient than the control group using the conventional Doppler ultrasound techniques. Our suggested model outperformed the existing techniques and is proved to be an effective method for early identification of the endometrial cancer using deep VGG-16 AdaBoost hybrid classifier.

## Figures and Tables

**Algorithm 1:**
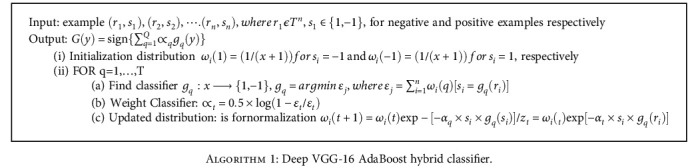
Deep VGG-16 AdaBoost hybrid classifier.

**Table 1:** Comparison of different algorithms for the control group.

Algorithm	Accuracy (%)	Sensitivity (%)	Specificity (%)	Kappa coefficient (%)
3D Doppler ultrasound using VOCAL [17]	75	74.2	60.6	83
Transvaginal ultrasound using logistic regression model [18]	80	85.71	83.67	60
Multivariate logistic regressions TUUS [19]	82	87.71	86.7	85
Deep VGG-16 AdaBoost hybrid classifier (proposed)	96.2	89	94.2	88

**Table 2:** Comparison of different algorithms for the experimental group.

Algorithm	Accuracy (%)	Sensitivity (%)	Specificity (%)	Kappa coefficient (%)
FIGO [20]	88	89.06	96.25	68.9
Contrast enhanced ultrasonography (CEUS) [21]	73	87	93	74.6
Transvaginal sonography [22]	86	89.8	90.9	76.6
DeepVGG-16 AdaBoost hybrid classifier (proposed)	98.6	91	97.8	81.5

## Data Availability

The analyzed datasets generated during the study are available from the corresponding author upon request.
